# Indirect Disaster‐Related Deaths Among Hemodialysis Patients Following the Fukushima Daiichi Nuclear Power Plant Accident

**DOI:** 10.1155/crin/8847100

**Published:** 2026-04-13

**Authors:** Ryoma Yoshida, Toyoaki Sawano, Yuna Uchi, Moe Kawashima, Kemmei Kitazawa, Hidenori Marui, Hiroki Yoshimura, Saori Nonaka, Naomi Ito, Chika Yamamoto, Toshiki Abe, Michio Murakami, Momoka Yamamura, Tianchen Zhao, Mamoru Sakakibara, Kazuko Yagiuchi, Mako Otsuki, Akihiko Ozaki, Tomoyoshi Oikawa, Shinichi Niwa, Masaharu Tsubokura

**Affiliations:** ^1^ Master’s Program in Medical Sciences, Graduate School of Comprehensive Human Sciences, University of Tsukuba, Ibaraki, Japan, tsukuba.ac.jp; ^2^ Department of Radiation Health Management, Fukushima Medical University, Fukushima, Japan, fmu.ac.jp; ^3^ Department of Surgery, Jyoban Hospital of Tokiwa Foundation, Iwaki, Japan; ^4^ Research Institute of Disaster Medicine, Chiba University, Chiba, Japan, chiba-u.ac.jp; ^5^ Department of Health Risk Communication, Fukushima Medical University, Fukushima, Japan, fmu.ac.jp; ^6^ Center for Infectious Disease Education and Research, The University of Osaka, Suita, Japan, osaka-u.ac.jp; ^7^ Reinstatement Support Center for Nurses, Incorporated Foundation of Tokiwa-kai, Iwaki, Japan; ^8^ St. Olive Nursing Home, Shirakawa, Japan; ^9^ Department of Nursing, Fukushima Medical University Hospital, Fukushima, Japan, fmu.ac.jp; ^10^ Breast and Thyroid Center, Jyoban Hospital of Tokiwa Foundation, Iwaki, Japan; ^11^ Department of Thyroid and Endocrinology, Fukushima Medical University, Fukushima, Japan, fmu.ac.jp; ^12^ Department of Neurosurgery, Minamisoma Municipal General Hospital, Minamisoma, Japan; ^13^ Department of Psychiatry, Aizu Medical Center, Fukushima Medical University, Aizuwakamatsu, Japan, fmu.ac.jp

## Abstract

**Introduction:**

Disaster‐related deaths (DRDs) are indirect fatalities caused by physical or psychological stress during evacuation. Patients undergoing hemodialysis (HD) face increased vulnerability during disasters due to reduced dialysis frequency, elevated mental stress, and limited access to medical resources. Although their risk is heightened, detailed analyses of DRDs in HD patients remain sparse.

**Methods:**

This retrospective study analyzed 13 HD‐related DRD cases in Minamisoma City, Fukushima Prefecture, following the Fukushima Daiichi Nuclear Power Plant accident. A total of 520 DRDs were certified in the city. Data from local government records were extracted, focusing on time of death, causes of death, and psychiatric symptoms.

**Results:**

The mean age (± standard deviation) at death for HD patients was 77.92 (±8.37) years, which was younger than the mean age (± standard deviation) of 82.81 (±11.97) years among non‐HD individuals (Welch’s *t*‐test, *p* = 0.060). Most deaths occurred during the chronic phase of the disaster. Primary causes included exacerbation of chronic kidney disease, cardiovascular complications, and sepsis. Over half of the patients exhibited psychiatric symptoms such as depression or mood instability.

**Discussion:**

This case series illustrates the severe impact of disruptions in medical care and the stress of repeated evacuations. Challenges include insufficient continuity of care and prolonged psychological distress, particularly during the chronic disaster phase. Our findings suggest that ensuring uninterrupted HD and providing long‐term psychological support may be essential to mitigating DRDs in this population.

## 1. Introduction

In Japan, the term “disaster‐related death” (DRD) is defined in the Act on Provision of Disaster Condolence Grant as deaths caused by the aggravation of injuries sustained in a disaster or diseases resulting from the physical burden of evacuation life [1]. The causes of DRDs are diverse. The criteria for certifying DRDs in Japan are determined at the local government level, although some local governments have published reference criteria, such as the Nagaoka Criteria, established after the 2004 Chūetsu earthquake, which classically certified deaths within 6 months of a disaster as DRDs [1]. Typical DRDs include respiratory and circulatory diseases caused by evacuation and limited access to medical facilities [1, 2]. According to the guidelines established by the Cabinet Office, individuals requiring nursing care and those with disabilities face an increased risk of death [3]. This heightened vulnerability is attributed to their potential inability to adapt to changes in their living environment, evacuation procedures, or the necessity of residing in evacuation shelters [4]. Despite the growing attention on DRDs in recent years, numerous challenges remain unaddressed, as available countermeasures are insufficient compared to those targeting direct deaths.

One prominent example of individuals highly susceptible to environmental fluctuations includes patients with chronic kidney disease (CKD), who are dependent on hemodialysis (HD) [5]. During disasters, patients requiring dialysis who are forced to evacuate may encounter physical burdens such as long‐distance travel and reduced dialysis frequency, as well as mental burdens associated with receiving treatment in unfamiliar and chaotic evacuation settings [3]. If these burdens persist, they can potentially impact the progression of the underlying disease [6, 7]. Previous studies on dialysis patients in disaster settings indicated that the scarcity of medical resources in the immediate aftermath of disasters may disrupt dialysis continuity for hospitalized patients [6]. Additionally, mental health complications have emerged as significant concerns for dialysis patients during disasters [8]. Moreover, a report has also noted notable blood pressure elevations following dialysis [9]. However, few reports exist on indirect DRDs among HD patients, and detailed descriptions of the processes leading to their death remain scarce.

The nuclear accident at the Fukushima Daiichi Nuclear Power Plant (FDNPP), caused by an earthquake and tsunami on March 11, 2011, led to widespread evacuations and severely impacted individuals vulnerable to environmental changes [10]. A study conducted in Minamisoma City, Fukushima Prefecture, which recorded the highest number of DRDs in the region, certified 520 DRDs by March 2022 [11, 12]. Research on dialysis patients after the FDNPP accident has noted issues such as blood pressure elevations during dialysis in the wake of the accident [13]. However, limited research exists on the prognosis of dialysis patients, including DRDs, following large‐scale evacuations.

In this study, from the dataset, we extracted cases of patients undergoing HD at the time of the disaster or who initiated HD as a result of evacuation, which included all DRD cases in Minamisoma City. These cases were analyzed with a focus on the time of death, causes of death, and the presence or absence of psychiatric symptoms. The study aimed to identify strategies to minimize and prevent DRDs among HD patients in the event of future radiation disasters.

## 2. Materials and Methods

### 2.1. Study Design and Setting

**FIGURE 1:**
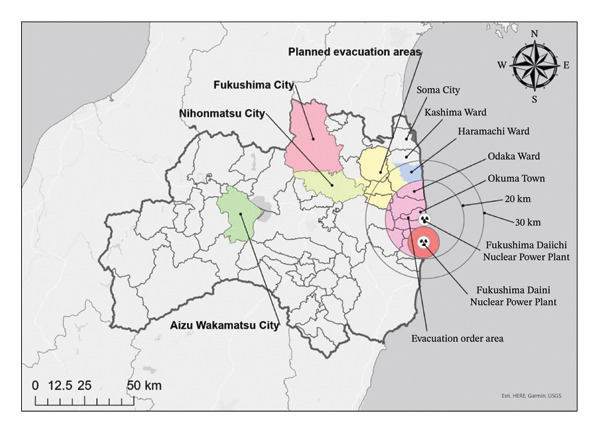
Location of Fukushima Daiichi Nuclear Power Plant and local governments involved in evacuation.

This study employed a retrospective observational design, focusing on residents of Minamisoma City, Fukushima Prefecture, whose deaths were certified as disaster‐related between March 11, 2011, and March 31, 2022. In the aftermath of the Great East Japan Earthquake, a tsunami resulted in the deaths of 636 individuals in Minamisoma. The southern region, encompassing most of Odaka Ward and part of Haramachi Ward, was designated as a restricted area [11]. In contrast, the central region, which includes a large part of Haramachi Ward, the southern section of Kashima Ward, and the northern tip of Odaka Ward, was categorized as an evacuation‐prepared area in the event of an emergency. The western part of Haramachi Ward has been classified as a deliberate evacuation area [11] (Figure 1). As of February 14, 2022, 520 deaths related to prolonged displacement have been recorded (Minamisoma City, 2022).

### 2.2. Study Participants

The study investigated 520 individuals residing in Minamisoma at the time of the earthquake, whose deaths were officially certified as disaster‐related by the Minamisoma City Committee for Certification of Disaster‐Related Deaths. In Japan, a DRD is defined as a fatality caused by the exacerbation of injuries sustained during the disaster or illnesses resulting from the physical strain of evacuation. These deaths are recognized under the Act on Provision of Disaster Condolence Grants (Act No. 82 of 1973), even in cases where no condolence payment was made [1]. However, this study excluded individuals whose whereabouts remained unknown after the disaster. Local governments certify DRDs by reviewing applications submitted by the deceased’s family [1]. Each municipality establishes its own certification committee to determine whether a death qualifies as disaster‐related. However, there are no unified national guidelines for the certification process, and public information regarding criteria or committee meeting frequency remains limited.

### 2.3. Data Collection

Records from the relevant local government committee in the disaster‐affected area were utilized to collect data on individuals certified as DRDs. Bereaved families submitted application forms and supporting documents, which were subsequently reviewed and summarized by the municipal office staff. Essential variables for analysis, including age, sex, date of death, residential location during the disaster, and reasons for certification, were extracted from these documents. To protect the privacy of the deceased and their families, all information was anonymized. The organized dataset was then prepared for statistical analysis to identify patterns and root causes of disaster‐related fatalities.

### 2.4. Data Extraction

We searched for the term “HD” in the DRD database of Minamisoma City, which contained information on the age and sex of the deceased, time elapsed between the disaster and death, residential location, and reasons for certification. Cases in which HD was being undergone at the time of the disaster or initiated due to evacuation were extracted and subjected to descriptive analysis.

### 2.5. Statistical Analysis

Continuous variables are presented as means ± standard deviations. Differences in mean age at death and the average duration from the disaster to death between individuals undergoing HD and those not undergoing HD were assessed using Welch’s *t*‐test, given the unequal sample sizes and variances between groups. All statistical tests were two‐sided, and a *p* value of < 0.05 was considered to indicate statistical significance.

### 2.6. Ethical Considerations

This study was approved by the Ethics Committees of Minamisoma Municipal General Hospital (approval number: 2–21) and Fukushima Medical University (reference number: 2020–297). The study was conducted using an existing administrative database of DRDs provided by the municipality. Information regarding the study was publicly disclosed using an opt‐out approach in accordance with institutional guidelines. The requirement for informed consent was waived because of the retrospective nature of the study and anonymization of the data. The study was conducted in compliance with the ethical principles outlined in the Declaration of Helsinki.

### 2.7. Case Presentation

A total of 520 individuals were classified as DRDs in Minamisoma City. Of these, 507 individuals not undergoing HD had a mean age (± standard deviation) of 82.81 (±11.97) years at death. In contrast, the deaths of 13 individuals undergoing HD were identified as DRDs (Table 1). Patients undergoing dialysis exhibited a lower mean age at death than those not undergoing HD, with a mean age at death of 77.92 (±8.37) years; however, this difference was not statistically significant (Welch’s *t*‐test, *p* = 0.060). Additionally, the average duration from the disaster to death for individuals not undergoing HD was 233.67 (±301.14) days, whereas those undergoing HD had a longer average duration of 500.31 (±506.98) days; however, this difference was not statistically significant (Welch’s *t*‐test, *p* = 0.073).

**TABLE 1:** Summary of the thirteen cases of disaster‐related deaths associated with hemodialysis.

No.	Direct cause of death	Sex	Age of death	Cause of death	Days from disaster to death	Mood swings or depression	Past medical history
1	Acute heart failure	Male	62	Acute heart failure	10	No	CKD, HD, Cerebral hemorrhage
2	CKD	Male	80	CKD	23	No	CKD, HD
3	Sepsis, disseminated intravascular coagulation syndrome	Female	80	CKD	54	Yes	Hypertension, CKD
4	CKD	Male	86	CKD	165	No	Right pleural effusion, DM, CKD HD, Hypertension, Anemia
5	Suffocation	Male	63	Suffocation	203	Yes	Cerebral infarction, DM, CKD, pulmonary disease, Heart disease
6	CKD	Female	81	Hepatic cirrhosis	275	No	Unknown
7	CKD	Male	72	DM	297	Yes	CKD, DM, Cerebral hemorrhage sequelae, Nephrosclerosis
8	Acute myocardial infarction	Male	86	Acute myocardial infarction	323	Yes	CKD, HD
9	CKD	Male	87	CKD	602	No	CKD, Hypertension, Chronic liver disease
10	Acute heart failure	Male	77	Acute myocardial infarction	670	Yes	Chronic CKD, HD, Brain hemorrhage
11	Sepsis	Female	83	Bilateral gangrene of lower extremity	1035	Yes	CKD, HD
12	Multiple organ failure	Female	84		1246	No	Chronic CKD, Hypertension Kidney anemia
13	CKD	Female	72	CKD	1601	Yes	CKD, Chronic heart failure, Peripheral artery disease

*Note:* HD, hemodialysis.

Abbreviations: CKD, chronic kidney disease; DM, diabetes mellitus.

### 2.8. Timeline of the Cases and Diagnostic Assessment

**FIGURE 2:**
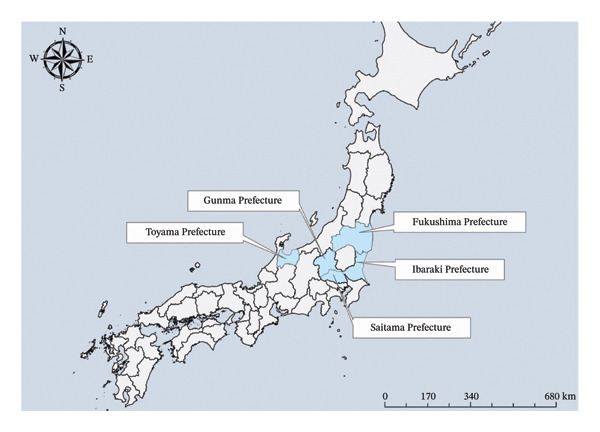
Location of prefectures where patients were evacuated.

1. A 62‐year‐old male with a history of CKD on HD and cerebral hemorrhage (with sequelae improved through rehabilitation) was hospitalized during the disaster owing to worsening kidney function. Following evacuation to another hospital, the patient experienced fatigue and exhaustion during transfer, leading to acute heart failure. He died of acute heart failure 10 days after the disaster.
2. An 80‐year‐old male with a history of CKD on HD was bedridden and undergoing HD three times weekly. After the disaster, he was hospitalized outside Fukushima Prefecture but could not continue HD after evacuation. HD was discontinued 6 days post‐disaster, and he was discharged. Returning to his home in Minamisoma City, his CKD worsened, resulting in death 23 days after the disaster.
3. An 80‐year‐old female with hypertension and CKD was undergoing daily peritoneal dialysis and weekly HD before the disaster. After the disaster, she evacuated to a relative’s house in the Soso District and was later hospitalized in Soma City. (Figure 1) Inadequate HD during the evacuation worsened her CKD, leading to sepsis and disseminated intravascular coagulation. She died 54 days after the disaster.
4. An 86‐year‐old male with a history of right pleural effusion, DM, CKD, hypertension, and anemia was receiving HD three times weekly. Following the disaster, he underwent five evacuations, which exacerbated his condition. He also experienced distress due to family separation and environmental changes. He died 165 days after the disaster at a hospital in Nihonmatsu City due to worsening CKD (Figure 1).
5. A 63‐year‐old male with a history of cerebral infarction, DM, CKD, heart disease, and lung disease was receiving HD three times weekly. The earthquake occurred during his HD, and he was subsequently evacuated to the Aizu Area, followed by relocation between hotels and temporary housing (Figure 1). Despite continuing HD at each location, his physical condition deteriorated, and he experienced ongoing anxiety. He was found suffocated in temporary housing in Fukushima City and was pronounced dead 203 days after the disaster (Figure 1).
6. An 81‐year‐old female with a history of liver cirrhosis and CKD on HD was hospitalized and underwent HD during the disaster. After being evacuated to a hospital in Toyama Prefecture, she continued on HD and was later transferred to a hospital in Fukushima Prefecture (Figure 2). However, owing to instability and inadequate treatment, her liver cirrhosis progressed. She died 275 days after the disaster due to worsening CKD.
7. A 72‐year‐old male with a history of CKD on HD, DM, cerebral hemorrhage sequelae, and nephrosclerosis was stable on dialysis three times a week before the disaster. After the disaster, he was transferred for surveillance and later hospitalized in Fukushima City (Figure 1). He contracted influenza, became debilitated, and required central venous nutrition due to difficulty in eating. Transfers and infection exacerbated his DM, and he died from CKD 297 days after the disaster.
8. An 86‐year‐old male with a history of CKD was undergoing HD three times a week at the time of the disaster. After evacuating to a shelter in Nihonmatsu City, he continued HD (Figure 1). However, after the shelter’s closure, he was transferred to a hospital in the Soso District (Figure 1). Physical exhaustion and mental stress led to an acute myocardial infarction at home following an HD session, and the patient died 323 days after the disaster.
9. An 87‐year‐old male with a history of CKD, hypertension, and chronic liver disease was receiving outpatient care for hypertension predisaster. Dialysis was not initially deemed necessary. After evacuating to Gunma Prefecture, he was hospitalized and discharged to temporary housing, where his kidney function worsened, leading to the initiation of HD (Figure 2). Following HD discontinuation due to poor health, his CKD worsened significantly, and he died 602 days after the disaster.
10. A 77‐year‐old male with a history of CKD and cerebral hemorrhage, who had used a wheelchair for daily living since a traffic accident 45 years earlier, was receiving HD three times a week via welfare taxi. After the disaster, he evacuated within Fukushima Prefecture, moving between shelters and relatives’ homes, undergoing seven relocations. He eventually settled in a rented house in Mito City, Ibaraki Prefecture, where he continued HD (Figure 2). He developed rectal bleeding, walking difficulties, and depression, frequently expressing a desire to return home. The patient suffered an acute myocardial infarction and died 670 days after the disaster from acute heart failure.
11. An 83‐year‐old female with a history of CKD was receiving outpatient HD before the disaster. After evacuating to a hotel in central Fukushima Prefecture, she continued HD at a local hospital. The lack of caregiving facilities at the hotel rendered her bedridden over time. She later relocated to her son’s home in Saitama Prefecture, where she continued HD (Figure 2). Her condition deteriorated owing to dementia, which led to gangrene in both her lower limbs. The patient died of sepsis 1035 days after the disaster.
12. An 84‐year‐old female with a history of CKD, hypertension, and renal anemia had been receiving regular outpatient care. After the disaster, she was unable to take medication for a week due to a hospital closure. Following a shunt procedure for HD and gastrostomy due to poor oral intake, her condition worsened. She died 1246 days after the disaster from multiple organ failure caused by hypoxemia.
13. A 72‐year‐old female with a history of CKD on HD, chronic heart failure, and arteriosclerosis obliterans was living independently predisaster. After evacuating to a relative’s home in Nakadori District, she struggled to find a hospital that could provide HD three times weekly (Figure 1). Her dementia rapidly progressed within a year postdisaster. Environmental changes, including the loss of social connections, caused mental instability, and she was diagnosed with schizophrenia. The patient was hospitalized in Fukushima City and died 1601 days after the disaster from worsening CKD (Figure 1).

## 3. Discussion

This report presents a detailed account of 13 certified DRDs in HD patients in Minamisoma City, Fukushima Prefecture, following the FDNPP accident. The findings reveal that the circumstances surrounding DRDs varied significantly based on the time elapsed since the disaster.

Most DRDs occurred during the chronic phase, more than 6 months after the disaster [14]. Of the 13 cases, only one occurred during the acute phase (within 1–2 weeks of the disaster), while four occurred within 6 months. In comparison, more DRDs during the Great East Japan Earthquake were reported within 6 months, suggesting a relatively higher proportion of dialysis patients certified as DRDs in the chronic phase in this study. Eight deaths occurred within a year of the disaster, while five occurred afterward. Notably, several of the cases that died within 6 months of the disaster (Cases 1–4) were attributed to worsening CKD or acute heart failure. These patients had a high probability of cardiovascular disease in addition to CKD, as indicated by their medical histories. Patients who died within 6 months to 1 year after the disaster (Cases 5–8) continued HD at evacuation sites but ultimately succumbed to worsening CKD or acute myocardial infarction. In cases where death occurred 1 year or more after the disaster (Cases 9–13), HD was maintained; however, the patients’ health gradually deteriorated, presumably as a result of prolonged evacuation, culminating in death from serious ailments such as heart failure, sepsis, multiple organ failure, or worsening CKD. Additionally, more than half of the patients (Cases 3, 5, 7, 8, 10, 11, and 13) exhibited symptoms of mood swings or depression. These findings indicate that risk factors contributing to mortality among HD patients after a disaster may vary depending on the disaster phase.

For individuals undergoing HD, the physical and psychological stressors associated with disaster evacuation may be more pronounced than those in the general population. Based on the clinical courses of the patients who died in the early phases of the disaster in our study, we postulate that the sudden onset of stress from repeated relocations and the demands of evacuation could elevate the risk of CKD deterioration or acute cardiovascular events. We further infer that this risk may be particularly pronounced in patients with cardiovascular disease and CKD. For this population, the physical strain of adapting to HD in unfamiliar facilities could be a contributing factor, potentially leading to increased blood pressure. This, in turn, places additional stress on arteries and kidneys, potentially exacerbating pre‐existing conditions. Furthermore, since physical activity is associated with reduced mortality risk among HD patients [15, 16], the decline in activity levels during evacuation [17] procedures may contribute to increased mortality risk in this population. Despite the passage of more than 6 months since the disaster and the ability to continue HD, the stress associated with the unfamiliar environment may persist. In such circumstances, a worsening of CKD or the occurrence of a vascular event could be a concern. Moreover, given that cases of depression (Cases 3, 5, 7, 8, 10, 11, and 13) often entailed multiple relocations, the physical and mental toll of moving and changing hospitals or clinics appeared to be substantial for these HD patients and may be an important factor to consider in the context of DRDs.

After more than 2 years of disaster‐related disruption, the inability to return to one’s original residence could also become a major source of psychological distress, particularly during prolonged evacuation. A prior survey revealed that 78.4% of disaster victims who evacuated from Okuma Town to Aizu Wakamatsu City exhibited signs of mental distress, a rate higher than that observed among other disaster victims [14] (Figure 1). This is attributed to prolonged stress from living in temporary housing and uncertainty regarding radiation decontamination [18]. We reason that this added physiological stress, which has been associated with elevated blood pressure [19] could, in turn, place additional strain on the circulatory system and kidneys, potentially increasing the risk of mortality in HD patients.

For some patients undergoing HD, evacuation may result in disruptions to HD access or inadequate coordination of care. Such disruptions could then result in insufficient treatment immediately after a disaster, a factor that may ultimately contribute to DRDs. Among the 13 cases presented here, four (Cases 3, 6, 11, and 13) are notable for the deterioration of the patients’ conditions and subsequent deaths in the context of inadequate HD following evacuation. These incidents can be attributed to the logistical challenges posed by the evacuation after the FDNPP accident. The influx of evacuees to designated hospitals strained resources, reducing the duration and frequency of HD sessions per patient. To prevent similar outcomes in the future, a disaster preparedness and response system specifically tailored to the needs of HD patients is essential. Predisaster measures should include collaboration between dialysis facilities and local governments to develop comprehensive evacuation plans, ensuring access to alternative facilities and transportation options. Regular disaster drills involving both patients and healthcare providers could enhance readiness and response capabilities. Based on the logistical challenges observed in our case series, we suggest that a centralized database for tracking the operational status of dialysis facilities could facilitate the efficient relocation of patients. We also propose that mobile dialysis units could provide temporary treatment in areas where fixed facilities are unavailable. Furthermore, we believe that government support to strengthen dialysis infrastructure, develop specialized equipment, and advance disaster‐specific medical technologies could enhance overall resilience. In our view, implementing such measures could significantly reduce DRDs among HD patients, ensuring their safety and well‐being during emergencies.

In the event of a prolonged disaster, psychological support may be necessary, particularly for patients undergoing HD. In this report, more than half of the patients (Cases 3, 5, 7, 8, 10, 11, and 13) exhibited depressive tendencies. These symptoms were observed in cases with an extended interval between the disaster and the patients’ deaths. Nevertheless, the prevalence of mental disorders among patients with end‐stage kidney disease undergoing HD is considerable [20]. As illustrated by several cases in our report, the psychological burden of treatment appears to be substantial. Our findings suggest that prolonged evacuation introduces additional stressors associated with environmental changes. We also observed that some patients lost conversation partners as a result of relocation. Therefore, we believe that active involvement from psychologists and psychiatrists is crucial to help in preventing declines in quality of life.

We propose a framework for considering HD care during disasters, dividing it into three phases: predisaster, acute phase, and chronic phase, to more effectively reduce DRDs among HD patients. In the predisaster phase, creating individualized emergency plans for patients and establishing systems for sharing patient information are essential [21, 22]. Following the Great East Japan Earthquake, over 1000 dialysis patients were classified as “refugees” in Fukushima Prefecture. Damage to medical facilities, equipment shortages, limited medical staff, and inadequate water and medicine posed significant challenges to maintaining life‐sustaining treatment [23]. This underscores the importance of conducting regular evacuation drills, developing contingency plans for securing essential supplies during hospital isolation, and maintaining durable HD equipment. In the acute phase, evacuation plans should leverage prior preparations. Research indicates that well‐planned evacuation saves vulnerable individuals’ lives [24]. We suggest that effective strategies could include matching patients to hospitals through information‐sharing systems, providing initial treatment at evacuation sites, prioritizing high‐risk patients for indoor treatment, ensuring appropriate HD frequency and duration, and reviewing meal content. We believe that implementing these measures minimizes physical exhaustion among HD patients and helps prevent DRDs during the acute and subacute phases of a disaster. In the chronic phase, accumulated mental stress from prolonged evacuation becomes a significant issue [14]. To mitigate this distress, it may be beneficial to cultivate an environment in which patients are less likely to experience feelings of isolation and can adapt more readily to their new circumstances. Based on the psychological distress observed in our cases, we suggest that consistent counseling and encouraging engagement with the local community could help alleviate distress and improve quality of life.

Our study concluded that in a dataset of DRDs in Minamisoma City, Fukushima Prefecture, following the nuclear accident, 13 of 520 DRDs involved patients undergoing HD. Except for one case, most deaths occurred during or after the chronic disaster phase, with the primary causes of death often recorded as the exacerbation of pre‐existing conditions such as CKD and cardiovascular disease. The clinical courses of these patients suggest that the health issues experienced by HD patients during disasters may be influenced not only by the physical stressors of the acute phase but also by the psychological stressors of the chronic phase caused by prolonged evacuation. Therefore, our findings emphasize the importance of providing long‐term psychological support after evacuation and ensuring uninterrupted HD care to reduce DRDs among HD patients during large‐scale disasters.

## Author Contributions

Ryoma Yoshida and Toyoaki Sawano: writing–original draft and writing–review and editing. Yuna Uchi, Moe Kawashima, Hidenori Marui, and Tianchen Zhao: data curation, formal analysis, and writing–review and editing. Kemmei Kitazawa: data curation and formal analysis. Hiroki Yoshimura, Saori Nonaka, Naomi Ito, Chika Yamamoto, Toshiki Abe, Michio Murakami, and Momoka Yamamura: writing–review and editing. Mamoru Sakakibara, Kazuko Yagiuchi, Mako Otsuki, and Tomoyoshi Oikawa: data curation and writing–review and editing. Akihiko Ozaki: writing–review and editing. Shinichi Niwa: conceptualization and writing–review and editing. Masaharu Tsubokura: conceptualization, data curation, and writing–review and editing.

## Funding

This study was supported by Japan Science and Technology Agency, JPMJPF2301.

## Disclosure

This study was supported by JST Grant Number JPMJPF2301 and the Network‐Type Joint Usage/Research Center for Radiation Disaster Medical Sciences.

## Ethics Statement

The research involving human participants received approval from the Ethics Committee of Minamisoma Municipal General Hospital (approval number: 2–21) and the Ethics Committee of Fukushima Medical University (reference number: 2020–297). The study procedures complied with all relevant local laws, guidelines, and institutional requirements. Written informed consent for participation was not required from the participants or the participants’ legal guardians/next of kin in accordance with the national legislation and institutional requirements.

## Conflicts of Interest

The authors declare no conflicts of interest.

## Data Availability

The data that support the findings of this study are available from Minamisoma City. Restrictions apply to the availability of these data, which were used under license for this study. Data are available from the author(s) with the permission of Minamisoma City.
